# Improved Cold Tolerance of Mango Fruit with Enhanced Anthocyanin and Flavonoid Contents

**DOI:** 10.3390/molecules23071832

**Published:** 2018-07-23

**Authors:** Pradeep Kumar Sudheeran, Oleg Feygenberg, Dalia Maurer, Noam Alkan

**Affiliations:** Department of Postharvest Science of Fresh Produce, Agricultural Research Organization, Volcani Center, P.O. Box 15159, HaMaccabim Road 68, Rishon LeZion 7505101, Israel; pradeepkumar2k@gmail.com (P.K.S.); fgboleg@volcani.agri.gov.il (O.F.); daliam@volcani.agri.gov.il (D.M.)

**Keywords:** anthocyanin, antioxidant activity, cold storage, ethylene, GC-MS, luminescence, multiplex, ROS

## Abstract

Red fruits were suggested to be tolerant to cold. To understand cold-storage tolerance of red mango fruit that were subjected to sunlight at the orchard, mango cv. Shelly from inside (green fruit) or outside (red fruit) the tree canopy was stored for 3 weeks at 5, 8 or 12 °C and examined for flavonoids, antioxidant, volatiles and tolerance to biotic and abiotic stress. Red fruit from the outer canopy showed significant increases in total anthocyanin and flavonoids, and antioxidant activity. Ripening parameters for red and green mango fruit were similar at harvest and during storage. However, red fruit with high anthocyanin and flavonoid contents were more tolerant to biotic and abiotic stresses. After 3 weeks of suboptimal cold storage, green fruit showed significantly more lipid peroxidation and developed significantly more chilling-injury symptoms—black spots and pitting—than red fruit. Volatiles of red and green peels revealed significant modulations in response to cold-storage. Moreover, red fruit were more tolerant to biotic stress and had reduced general decay incidence. However, during long storage at 10 °C for 4, 5 or 6 weeks, red fruit showed a non-significant reduction in decay and chilling injuries. These results suggest new approaches to avoiding chilling injury during cold storage.

## 1. Introduction

Mango (*Mangifera indica* L.), is an economically important fruit, distributed worldwide, which belongs to the family *Anacardiaceae*. Fully ripe mango is known for its aroma, peel color, good taste and nutritional value [1,2]. It is also highly perishable; soon after ripening, the fruit starts decaying and quickly becomes unfit for consumption. Cold storage is probably the best postharvest technology to maintain high-quality fruit [3]. However, as a tropical fruit which was not exposed to extreme cold temperature during its evolution, mango is highly susceptible to cold storage. Storage of mango fruit at temperatures lower than 12 °C leads to the development of chilling injuries, which are expressed as physiological and biochemical alterations and cellular dysfunctions. These alterations include stimulated ethylene production, increased respiratory rate, enzyme inactivation, membrane dysfunction, production of reactive oxygen species (ROS) and changes in cellular structure, which lead to the development of chilling-injury symptoms such as pitting, black spots, peel discoloration, water-soaked appearance, internal breakdown and browning, uneven ripening, off-flavor and decay [4,5]. This limits the application of cold storage to extend mango’s lifetime. Recently, mango fruit transcriptome was characterized in response to storage at suboptimal temperature and showed activation of defense response signal transduction, lipid peroxidation and a significant activation of the phenylpropanoids biosynthetic pathway [6].

Anthocyanins are phenylpropanoids that are widely distributed in vascular plants where they serve as inducible sunshields [7]. Polyphenols, like anthocyanins and flavonols, are one of the mango fruit’s antioxidant compounds [8]. In mango fruit peel, anthocyanins are responsible for the red color, while carotenoids are responsible for the yellow to orange color [9].

Anthocyanins’ antioxidant capacity makes them important phytonutrients in a healthy diet, along with their antitumor, anti-inflammatory and antineurodegenerative properties [10,11]. Both anthocyanins and flavonols are products of the phenylpropanoid biosynthetic pathway and are involved in plant protection against pathogens [12]. Indeed, when 83 mango cultivars were infected with *Colletotrichum gloeosporioides* or stored at suboptimal temperature of 6 °C, the red mango cultivars showed a significant increased tolerance to both anthracnose caused by *C. gloeosporioides* and to chilling injuries after storage in comparison to green cultivars [13].

Anthocyanin production can be induced by abiotic stress. Anthocyanins and flavonols are known to increase in response to cold [14,15]. Another environmental cue regulating anthocyanins is light. Indeed, mango fruit that are exposed to sunlight in the orchard accumulate anthocyanins and a red skin color [12].

This manuscript’s aim is to characterize and study the cold tolerance mechanism of red mango fruit that contain high anthocyanin and flavonol contents in order to store red mango fruit at lower temperature and to extend its storage period.

## 2. Results

### 2.1. Evaluation of Mango Fruit Color, and Anthocyanin and Flavonoid Contents

Mango (*Mangifera indica* L., cv. Shelly) fruit were harvested from two different positions on the tree canopy: red-colored fruit from the exterior position with direct exposure to sunlight, and green-colored fruit from the interior position, in the shaded part of the canopy. Uniform, unblemished fruit were selected on the basis of skin color: red fruit with more than 60%, and green fruit with less than 10%, of the fruit peel colored in red. The fruit were stored in the cold at 5, 8 or 12 °C for 3 weeks and transferred to 1 week of shelf life at 20 °C.

**Figure 1:**
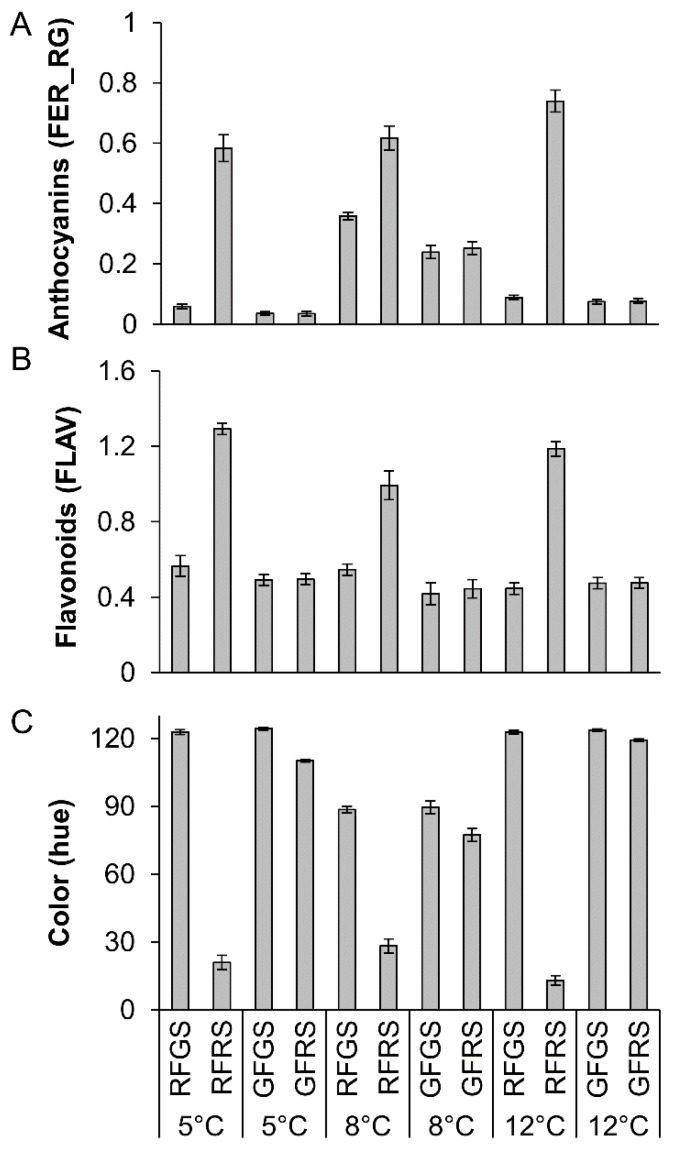
Quantification of color, anthocyanins and flavonols by fluorescence and light-based methods in the peel of red mango fruit (RF) or green fruit (GF) on their red side (RS) or green side (GS) after cold storage at 5 °C, 8 °C or 12 °C. (**A**)Total anthocyanin content. (**B**) Total flavonolcontent. (**C**) Fruit color displayed as hue.

Mango skin color was measured after cold storage and presented as hue. Both the green fruit and the red fruit on their green side had similar values (100–120) correlated to green color, while the red side of the red fruit presented hue values of 13–28, correlated to red color (Figure 1). Using a Multiplex III fluorescence detector, we measured the fruit fluorescence and assessed different chemical groups as flavonoids (FLAV) and anthocyanin (FER_RG) [15]. The FER_RG and FLAV ratio measurements, representing anthocyanin and flavonoids, revealed that the red side of the red fruit had about 3-fold higher flavonoid content about 10-fold higher anthocyanin content than the red side of the green fruit after cold storage (Figure 1). Similar results were found for extended storage (see Section 2.6) at 10 °C (Figure S1). Interestingly, after storage of ‘Shelly’ mango fruit at 8 °C, a minor change of color was observed on both the green side of the red fruit and in the green fruit (Figure 1C), correlated to an increase in anthocyanin content in the peel of the green fruit after cold storage (Figure 1A).

**Figure 2:**
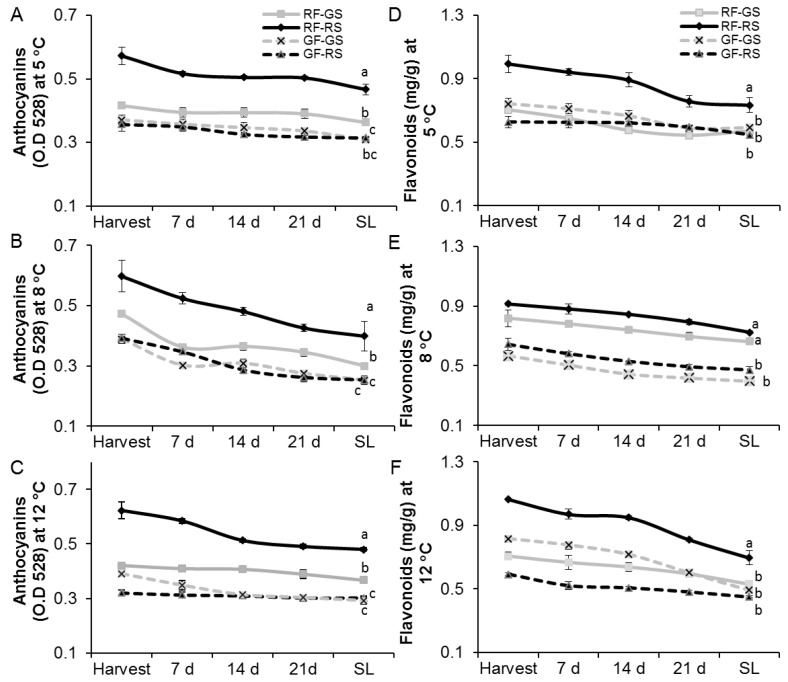
Quantification of total anthocyanins and flavonoids by chemical methods in the peel of red mango fruit (RF) or green fruit (GF) on their red side (RS) or green side (GS). (**A**)Total anthocyanin content during cold storage at 5 °C. (**B**) Total anthocyanin content during cold storage at 8 °C. (**C**) Total anthocyanin content during cold storage at 12 °C. (**D**) Total flavonoid content after cold storage at 5 °C. (**E**) Total flavonoid content after cold storage at 8 °C. (**F**) Total flavonoid content after cold storage at 12 °C. SL, shelf life. Average and SE are presented. Different letters indicate significant difference (*p* < 0.05).

Total amount of anthocyanins and flavonoids was estimated chemically for red and green fruit stored at 5, 8 or 12 °C for 3 weeks and an additional 7 days of shelf life (Figure 2). A significant 2-fold increase in the amount of anthocyanins was seen on the red side of the red fruit compared to both red and green sides of the green fruit (Figure 2A–C). While the green side of the red fruit showed only a moderate level of anthocyanin, it was still significantly higher than that in the green fruit. In all, the different cold-storage conditions showed similar amounts of anthocyanin (Figure 2A–C). In addition, contents of both anthocyanin and flavonols declined moderately, by about 20%, during storage and as the fruit ripened. Similarly, the amount of flavonoids was significantly higher on the red side of the red fruit compared to both sides of the green fruit (Figure 2D–F), whereas storage at different temperatures did not affect the flavonoid content in the mango peel (Figure 2D–F). Thus, both anthocyanins and flavonoids were accumulated mostly at the red side of the red fruit and gradually decreased during storage.

### 2.2. Antioxidants and ROS

**Figure 3:**
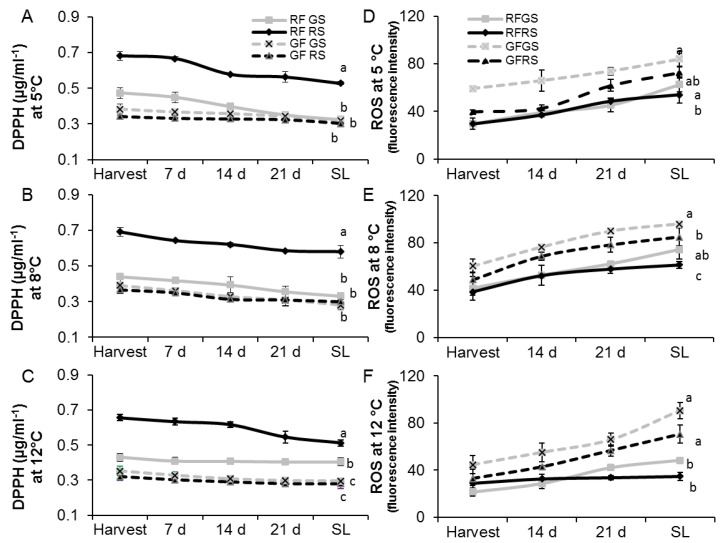
Antioxidant activity and ROS in the peel of red mango fruit (RF) or green fruit (GF) on their red side (RS) or green side (GS). (**A**–**C**) Antioxidant activity at different storage temperatures (5, 8 and 12 °C, respectively). (**D**–**F**) Reactive oxygen species (ROS), as quantified by relative fluorescence intensity, in red and green peel of ‘Shelly’ mango stored at 5, 8 or 12 °C, respectively. SL, shelf life. Average and SE are presented. Different letters indicate significant difference (*p* < 0.05).

To measure the antioxidant activity of flavonols and anthocyanins in red and green peels of mango fruit, radical-scavenging activity of DPPH was assessed in peels of fruit stored at 5, 8 or 12 °C for 3 weeks and an additional 7 days of shelf life. A significant 2-time increase in the amount of scavenging activity of DPPH was seen on the red side of the red fruit compared to both sides of the green fruit (Figure 3A–C). However, no significant differences were observed among the different temperature treatments (Figure 3A–C). The increase in antioxidant activity of the red fruit on its red side was well correlated to the increase in flavonoids and anthocyanins (compare Figure 2 and Figure 3). Thus, the red fruit accumulated anthocyanins, flavonoids and antioxidant activity mostly at their red side.

Accumulation of ROS was detected by fluorescence microscopy after DCF staining of mango peels stored at 5, 8 or 12 °C for 3 weeks and an additional 7 days of shelf life. The ROS fluorescence intensity was significantly increased (2-fold) in the green peel compared to the low fluorescence and ROS levels detected in the red peel (Figure 3D–F). It was also noted that the relative ROS levels increased with time from harvest to cold storage to shelf life under all temperature regimes (Figure 3D–F), and this increase was negatively correlated to the reduction of antioxidant activity during storage and fruit ripening (Figure 3A–C).

### 2.3. Chilling Tolerance of Red Mango Fruit

**Figure 4:**
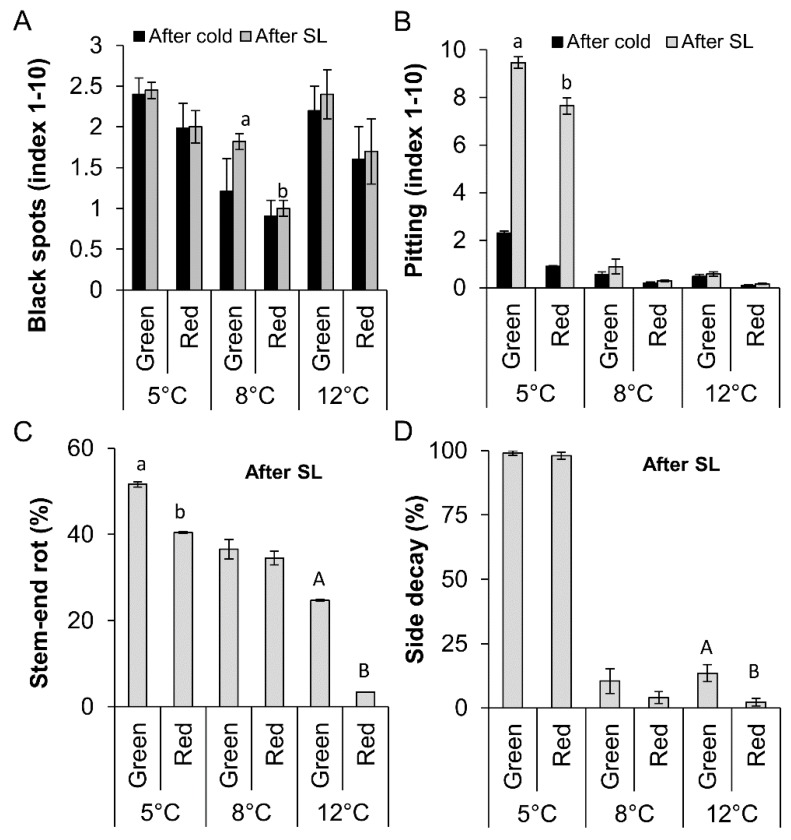
Evaluation of chilling injuries of red and green mango fruit after 3 weeks of cold storage (5 °C, 8 °C or 12 °C) and a further 7 days of shelf life (SL) (20 °C). (**A**) Black spot severity (index 1–10). (**B**) Pitting severity (index 1–10). (**C**) Incidence of stem-end rot after SL in red and green fruit. (**D**) Incidence of side decay after SL in red and green fruit. Average and SE are presented. Different letters indicate significant difference (*p* < 0.05).

Chilling tolerance of red mango fruit was assessed on the basis of severity of black spots and pitting (scale of 0–10) in red and green mango fruit after 3 weeks of cold storage at 5, 8 or 12 °C and an additional 7 days of shelf life at 20 °C. As expected, fruit stored at the suboptimal temperature of 5 °C had more chilling injuries, expressed as black spots and pitting, than fruit stored at higher temperatures. However, red fruit had less black spots and pitting damage than green fruit after cold storage and after additional shelf life (Figure 4A,B), whereas more red spots were observed on red vs. green fruit (data not shown).

Natural stem decay and side decay were evaluated after cold storage at the different temperatures and additional shelf life. After shelf life, when the fruit ripened, a correlation was found in which fruit stored at lower temperature developed more stem-end rot than those stored at higher temperature (Figure 4C,D). Furthermore, red fruit had less decay incidence than green fruit (Figure 4C). Likely, due to the severe pitting induced upon cold storage at 5 °C, a higher incidence of side decay was found in fruit stored at 5 °C than in those stored at higher temperatures (Figure 4B,D).

### 2.4. Lipid Peroxidation during Cold Storage

**Figure 5:**
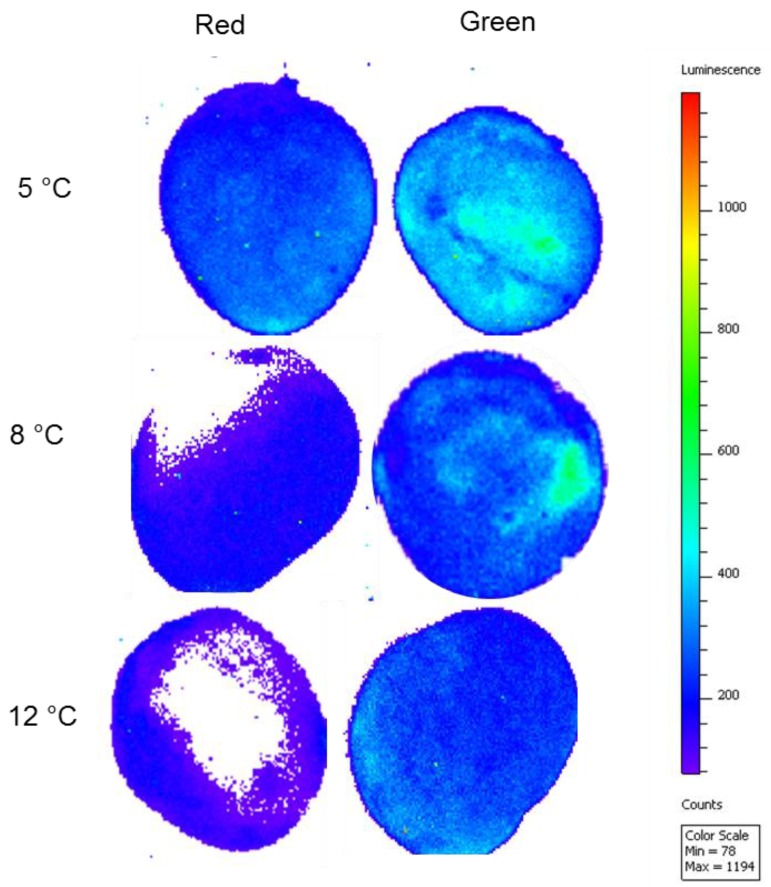
Mango fruit luminescence. Evaluation of luminescence and lipid peroxidation in red and green mango fruit after 3 weeks of cold storage at 5 °C, 8 °C or 12 °C by *in-vivo* imaging system (IVIS). Luminescence of lipid peroxidation of red fruit (left panels) and green fruit (right panels) after 3 weeks storage at 5 °C (top panel), 8 °C (middle panel) and 12 °C (bottom panel).

Lipid peroxidation is known to occur with chilling injuries and can be detected by bioluminescence [6]. During cold storage of red and green mango fruit, the luminescence of the whole fruit was measured with an *in-vivo* imaging system (IVIS). The fruit stored at lower temperatures (5 or 8 °C) showed more luminescence than those stored at the optimal temperature of 12 °C (Figure 5). Interestingly, green fruit had considerably more chilling injury symptoms (Figure 4A,B) and more luminescence than the red fruit stored at suboptimal temperatures (Figure 5). Indeed, red fruit stored at 8 °C showed very low luminescence, which was correlated with low levels of lipid peroxidation and chilling injury.

### 2.5. GC–MS Analysis of Mango Volatiles

**Figure 6:**
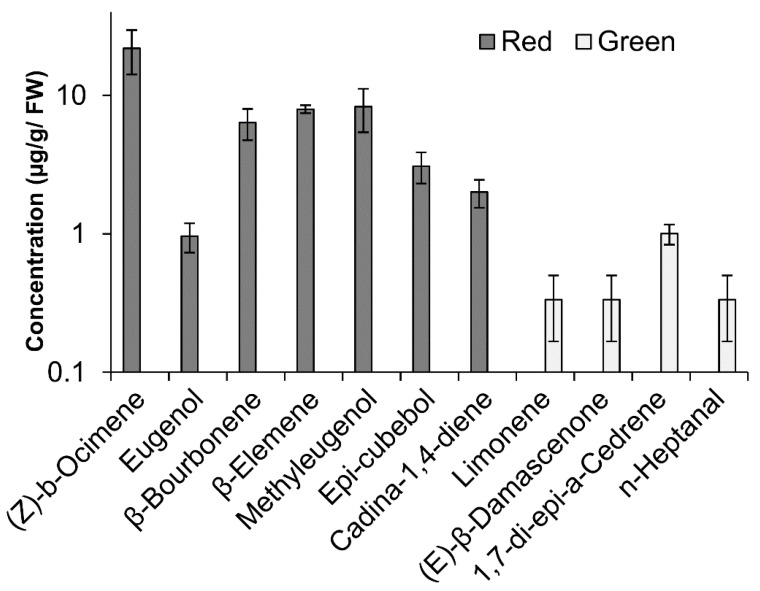
GC-MS analysis of volatile compounds of red and green peels of ‘Shelly’ mango after 3 weeks of cold storage at 12 °C. Graphical representation of the concentration of unique volatiles of red and green mango peel.

Volatile compounds were identified in the peel of red and green ‘Shelly’ mango fruit after 3 weeks of cold storage; a total of 28 putative volatile compounds were identified and quantified using calibration graph and internal standard (Table S1). Among them, 11 volatiles were significantly altered during cold storage of red fruit compared to green fruit (Figure 6). Four volatile compounds (limonene, (E)-β-damascenone, 1,7-di-epi-α-cedrene, *n*-heptanal) were upregulated in green fruit peel, and found in a concentration of 0.3–1 µg/gFW, and were not detected in the peel of red fruit. While, seven volatile compounds ((*Z*)-β-ocimene, eugenol, β-bourbonene, β-elemene, methyleugenol, epi-cubebol, cadina-1,4-diene) were elevated in the red fruit peel and found in a concentration of 0.9–21 µg/gFW and were not detected in the peel of the green fruit (Figure 6). Interestingly, compounds as eugenol and methyleugenol that were increased in red fruit peel are products of the phenylpropanoid pathway, while other compounds are not. Thus, a significant change in volatiles profile was observed between the peels of red and green mango fruits.

### 2.6. Storage Elongation

To increase mango fruit storage, red and green fruits were stored at 10 °C (instead of the optimal 12 °C) for 4 weeks, 5 weeks or 6 weeks (instead of the usual 3 weeks). Then the fruit were transferred to 1 week of shelf life at 20 °C. Both red and green fruit showed more or less similar ripening parameters during storage, including: (i) decreasing firmness; (ii) increasing total soluble solids (TSS); and (iii) decreasing citric acid content (Figure S2). Supporting the similar ripening of red and green mango fruit, ethylene and respiration (CO_2_) were similar after 3 weeks of cold storage at 5, 8 or 12 °C (Figure S3). Interestingly, while the ripening parameters were relatively similar, the red fruit had higher TSS and lower acid concentration than the green fruit after extended cold storage and additional shelf life (Figure S2)**.** These increased sugars and decreased acids are correlated to better mango fruit quality and taste. A similar increase in TSS and reduction in acids was recorded in red vs. green mango fruit stored at 12 °C (Figure S4).

**Figure 7:**
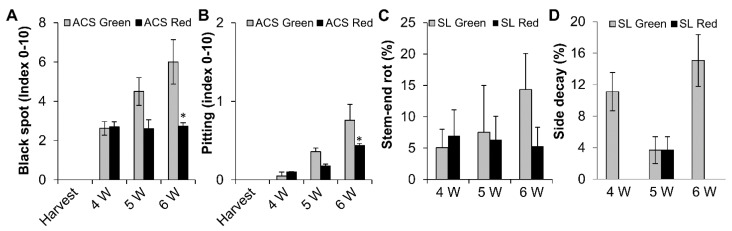
Postharvest chilling and decay in red and green ‘Shelly’ mango fruit stored at 10 °C for 4–6 weeks (W). (**A**) Black spot (index 0–10) after cold storage (ACS) at 10 °C for 4, 5 or 6 weeks. (**B**) Pitting (index 0–10) after cold storage at 10 °C for 4, 5 or 6 weeks. (**C**) Stem-end rot incidence (percentage) after cold storage at 10 °C for 4, 5 or 6 weeks and further shelf life (SL). (**D**) Side decay incidence (percentage) after cold storage at 10 °C for 4, 5 or 6 weeks and further shelf life.

As expected, red fruits showed fewer chilling injuries, expressed as black spots and pitting, after 5 and 6 weeks of cold storage (Figure 7A,B). Moreover, red fruit showed a non-significant reduction in natural postharvest stem-end rot after long storage (Figure 7C), whereas a significant reduction was observed in natural side decay of red fruit compared to green fruit after 4 and 6 weeks of storage (Figure 7D). Thus, red fruit had less chilling injuries and postharvest decay and could be stored for longer periods of time, as demonstrated by representative pictures (Figures S5 and S6).

## 3. Discussion

In response to light, ‘Shelly’ mango fruit that grew on the outer part of the tree canopy accumulated anthocyanin and red color in their peel, whereas the non-exposed surfaces of red fruit or fruit that developed inside the canopy were less exposed to sunlight and remained green [12]. Both anthocyanin and flavonols have pleotropic effects and are known to be involved in plant protection against pathogens [16,17].

In this study, ‘Shelly’ mango fruit were harvested from outside (red fruit) and inside (green fruit) the tree canopy. Analysis of total content of flavonoids and anthocyanin showed that both increased by 2-fold in the red mango fruit on their red side, which was exposed to sunlight (Figure 1). These results suggest that exposure to light increases the phenylpropanoid pathway in mango fruit leading to increased flavonols and anthocyanin. Anthocyanin is induced by a number of environmental factors, including high light, UV radiation, cold temperatures and water stress, and it has been proposed as an important compound in the plant’s response to abiotic stress [18,19,20].

The storage temperature (5, 8 or 12 °C) did not affect the flavonoid or anthocyanin levels in the mango fruit. However, both flavonol and anthocyanin levels gradually decreased with time of storage.

Both flavonols and anthocyanins are known to have antioxidant activity [21]. As expected, red fruit had 2- to 3-time increased antioxidant activity compared to green fruit (Figure 3). Indeed, studies have shown that tomato *Delila* and *Rosea1* mutants accumulate anthocyanins, resulting in reduced ROS accumulation and reduced susceptibility to gray mold [22]. Similarly, in the present study, both sides of the red fruit had reduced ROS levels at harvest relative to green fruit (Figure 3). Exposure of plants to low temperatures can cause an increase in ROS production in various plant tissues [1]. Accordingly, ROS were accumulated during cold storage, and more significantly at suboptimal temperature (5 or 8 °C). However, ROS accumulation in response to cold storage was inhibited on both sides of the red fruit as compared to green fruit (Figure 3). This reduction in ROS was tightly correlated with the high antioxidant activity in the red mango fruit, which had higher levels of anthocyanins and flavonols (Figure 2 and Figure 3).

ROS accumulation during storage at suboptimal temperature activates lipid peroxidation, which is a main characteristic of initial chilling injuries in mango fruit [5]. This lipid peroxidation can be visualized by bioluminescence [5]. As expected, fruit stored at lower temperature had higher luminescence. Furthermore, green fruit, with higher levels of ROS, showed a 2- to 3-timeincrease in luminescence and lipid peroxidation compared to the red mango fruit (Figure 5). The reduced lipid peroxidation in red mango fruit was correlated with reduced chilling injuries in these fruit (Figure 4). Thus, we suggest that the mechanism of increased tolerance of red mango fruit to chilling injury involves higher antioxidant activity and high levels of anthocyanin and flavonols that lead to reduced ROS in response to cold storage; this, in turn, leads to reduced lipid peroxidation and reduced chilling injuries. Alternatively, exposure of the red fruit to direct sunlight in the orchard might induce fruit resistance to chilling injuries during storage.

After cold storage at optimal temperature (12 °C), 28 putative volatile compounds of mango fruit [23,24] were detected. However, the overall volatile profile of the red fruit was significantly different from that of the green fruit in response to cold storage, showing seven unique compounds in red fruit and four unique compounds in green fruit (Figure 6). As expected, several of the volatiles that were unique to red fruit, such as eugenol and methyleugenol, are products of the phenylpropanoid pathway, which is known for conferring a good flavor, and antioxidant and anti-infective activity [25]. In the present study, the major compounds detected in the peel of red mango cv. ‘Shelly’ were (*Z*)-β–ocimene, β-elemene, epi-cubebol, cadina-1,4-diene and β-bourbonene, which are known as aroma volatiles of monoterpenes and sesquiterpenes. Those volatiles were observed in previous studies on different mango cultivars and their volatile concentration were in a similar range. For example, volatiles as cadina-1,4-diene were observed in cv. ‘Paulista’, Epi-cubebol in cv. ‘Espada’, β –ocimene in cv. ‘Espada’, β-elemene in cv. ‘*Coquinho*’ and β-bourbonene in cv. ‘Nam Dok Mai’ [26,27,28]. Interestingly, all of those volatiles compounds are known to have antifungal and antibacterial activity [29,30,31]. Red mango fruit, which were exposed to light and accumulated anthocyanin, were more tolerant to postharvest chilling. To examine longer storage times, mango fruit were stored at 10 °C for 4–6 weeks instead of the common practice of 3 weeks’ storage at 12 °C. The red and green fruit, picked from the same orchard on the same day, showed no significant difference in ripening parameters: all of the fruit turned yellow, and showed increased TSS and reduced citric acid as storage and ripening progressed (Figure S2). Interestingly, the red fruit had a higher level of TSS and lower acidity after shelf life. This is probably because the red fruit were exposed to light during their growth and accumulated higher levels of starch. Increased sugar and reduced acidity are correlated to better taste and acceptance [3].

While both red and green mango fruit presented similar ripening progress, fruit with a high percentage of anthocyanin and red color in their peel were more resistant to chilling injuries and presented less black spots and pitting (Figure 7). Our previous study suggested that black spots and pitting in mango fruit are actually discolored lenticels that accumulate dead cells in response to chilling [32]. This programmed cell death was correlated with the increase in ROS. A similar increase in ROS was seen in green mango stored at suboptimal temperatures (Figure 3). Interestingly, as chilling injuries is due to discolored lenticels accumulated in the green mango, more natural opening occurred. This natural opening could be a suitable penetration site for pathogenic fungi [6]. Indeed, more side decay was observed in the green fruit that had more black spots and pitting. 

To summarize, this study showed that ‘Shelly’ mango fruit exposed to sunlight in the orchard activate the phenylpropanoid pathway and accumulate flavonols and anthocyanin. Accumulation of these flavonoids was correlated with increased antioxidant activity and reduced ROS, leading to reduced lipid peroxidation, thereby enhancing the fruit’s tolerance to chilling during storage at suboptimal temperature. Following these results, red mango fruit stored at 10 °C had reduced chilling injury and reduced decay and could be stored for longer periods of time than the green fruit. This manuscript findings should result in a future applicative research that will induce the accumulation of anthocyanins in the peel of mango fruit in order to modify the fruit to a more attractive and tolerant fruit to both biotic and abiotic stress.

## 4. Materials and Methods

### 4.1. Plant Material and Storage Conditions

Mango (*Mangifera indica* L., cv. Shelly) fruit were harvested in July 2016 and 2017. Fruits were picked from two different canopy positions in the orchard: the red-colored fruit from the exterior position with direct exposure to sunlight, and the green-colored fruit from the interior position in the shaded part of the canopy. Five hours after harvest, the fruit were transported from ‘Mor-Hasharon’ storage house, Israel to the Agricultural Research Organization, Volcani Center, Israel (1 h transport time). Uniform, unblemished fruit weighing approximately 400 g were selected on the basis of peel color: red fruit had more than 60% of the fruit peel colored in red and green fruit had less than 10% of the fruit peel colored in red. The fruit were washed with tap water and air-dried, and then stored at 5, 8 or 12 °C for 3 weeks in the cold-storage rooms. Each treatment consisted of 6 cardboard boxes with 10–12 fruits in each. After cold storage, the fruit were stored for 7 days at 20 °C to mimic shelf life. In the second experiment, red or green mango fruit were stored at 10 °C for 4 weeks, 5 weeks or 6 weeks and then transferred to 20 °C for 7 days to mimic shelf life. Each treatment consisted of six cardboard boxes with 10–12 fruits in each. The temperature in the cold-storage room was monitored by a DAQ tool—double-strand wire logger/data acquisition control system (T.M.I. Barak Ltd., Ramat Gan, Israel). Each experiment was repeated in the years 2016 and 2017.

### 4.2. Evaluation of Physiological Parameters of Red and Green Mango Fruit

Ripening and physiological parameters—firmness (Newton), TSS, and acidity (citric acid equivalence)—of red and green mango fruit were measured at harvest, after cold storage and after the additional 7 days of shelf life. Fruit firmness was determined by an electronic penetrometer force gauge LT-Lutron FG-20 KG (Jakarta, Indonesia) with an 11-mm probe at two points on the equatorial line of each fruit (20 measurements per treatment). Percentage of TSS was measured from the juice of fruit pulp using Palette digital-refractometer PR-1 (Model DBX-55, Atago, Tokyo, Japan), 10 fruits per treatment. The acidity was determined as citric acid equivalent mass in 1 mL of pulp juice of red and green mango that was dissolved in 40 ml of double distilled water, using an automatic titrimeter (Model 719 s, Titrino Metrohm Ion Analysis Ltd., Herisau, Switzerland), 10 measurements per treatment.

### 4.3. Mango Fruit Physiological and Pathological Parameters

Physiological and pathological parameters of the mango fruit were assessed on the basis of severity of black spots, red spots, pitting, stem decay and side decay in red and green mango fruit after cold storage (5, 8 or 12 °C) and a further 7 days of shelf life at 20 °C. Black spot and red spot severity indices were evaluated on a relative scale of 0–10 (1 representing mild black spots and 10 representing severe black spots, 40 evaluations per treatment). Pitting severity index was presented on a relative scale of 0–10 (1 representing mild pitting and 10 representing severe pitting, 40 evaluations per treatment). Stem decay and side decay incidence was evaluated as percentage of decayed red or green mango fruit in each box.

### 4.4. Analysis of Peel Color of Red and Green Mango Fruit

The mango peel color was measured after harvest, after cold storage and after shelf life using a chromometer model CR-400/410 (Konicka Minolta, Osaka, Japan) at two points on the equatorial line of each fruit, at the red side and at the green side (20 measurements per treatment) and presented as hue angle, where 0 = red, 60 = yellow, 120 = green. Anthocyanin and flavonoid contents were measured in a Multiplex III fluorescence detector (Force A, Orsay, France), consisting of 12 fluorescent signals. The ratios between these signals in different mathematical expressions were correlated to the fluorescence of major chemical groups, e.g., anthocyanin (FER_RG, the ratio of far-red emission excited by red or green light) and flavonoids (FLAV, the logarithm of the red to ultraviolet [UV] excitation ratio of far-red chlorophyll fluorescence) [33]. The methodology was as described in Bahar et al. [33]. Ten fruit were measured from both sides for each treatment after harvest and after cold storage.

### 4.5. Determination of Total Anthocyanin and Flavonol Contents in Red and Green Mango Fruit

Total anthocyanin content was determined for peel extract of red and green mango fruit stored in the cold (5, 8 or 12 °C) for 3 weeks and an additional 7 days of shelf life by spectrophotometry after organic methanol extraction of mango fruit peel and further measurement of absorption at 528 nm [34]. The total flavonoid content in the red and green mango peel was extracted and measured for fruits stored under different cold-storage conditions (5, 8 or 12 °C) for 3 weeks and an additional 7 days of shelf life using the aluminum chloride colorimetric method [35] and different concentrations of quercetin was used as a standard for quantification. The absorbance was measured at 415 nm using a UV spectrophotometer, and the levels of total flavonol contents were determined in triplicates, respectively. Results were expressed as mg quercetin in 1 g of sample (mg QE/g).

### 4.6. DPPH Radical-Scavenging Capacity

The DPPH radical-scavenging activity of red and green mango peel extracts was estimated according to the method followed by Cheung et al. [36] with slight modifications. In this assay, antioxidants present in the sample reduced the DPPH radicals, which absorb at 517 nm. Several concentration of ascorbic acid was used as a reference standard for quantification, the reaction was carried out in triplicate. The ‘Shelly’ mango peel was extracted following different cold-storage conditions (5, 8 or 12 °C for 3 weeks) and an additional 7 days of shelf life.

### 4.7. Analysis of ROS Production and Confocal Microscopy

Mango fruit peel (200-µm thickness) was taken from red fruit–red side and green side, and green fruit–red side and green side, and incubated with 10 µM of 2,7-dichlorodihydro fluorescein diacetate (H_2_DCF-DA) in phosphate buffered saline (PBS) for 15 min in the dark, then washed twice with PBS. The stained peels were observed under fluorescence microscope (Olympus-BX53, Tokyo, Japan) using GFP3 excitation and emission wavelengths. The relative intensity of the fluorescent signal was calculated, using Image J software, as the average intensity from three focal planes in three biological repeats for each sample of red or green sides of the red or green fruit stored at 5, 8 or 12 °C for 3 weeks and an additional 7 days of shelf life.

### 4.8. Evaluation of Lipid Peroxidation by IVIS

Red and green mango fruit were randomly selected after 3 weeks of cold storage at 5, 8 or 12 °C to detect lipid peroxidation level using a pre-clinical IVIS (PerkinElmer, Waltham, MA, USA). Fruit were kept in the dark for 2 h prior to evaluation. Lipid peroxidation in the fruit was detected and visualized by auto-luminescence of peroxide lipids as described previously [6], using auto-luminescence for 20 min with emission at 640–770 nm. The auto-luminescence was recorded with a highly sensitive charge-coupled-device (CCD) camera. The optical luminescent image data are presented as intensity in terms of radiance (photons^−1^ cm^−2^ steradian). The measurements were repeated three times with different fruits.

### 4.9. GC-MS Analysis of Mango Volatiles

Peel (1 g) was sampled from seven red and seven green ‘Shelly’ mango fruit for each replicate, and three biological replicates were collected. Each replicate was collected in a 20-mL amber vial (LaPhaPack, Langerwehe, Germany) prepared in advance with 5 mL of 20% (*w*/*v*) NaCl (Sigma-Aldrich, St Louis, MO, USA), 0.6 g NaCl, and 25 μL of 10 ppm S-2-octanol (Sigma-Aldrich) added as an internal standard. On the day of analysis, samples were prewarmed for 1 h at 30 °C on an orbital shaker at 250 rpm.

A solid-phase microextraction (HS-SPME) holder (Agilent, Palo Alto, CA, USA) assembled with a fused silica fiber (Supelco, Bellefonte, PA, USA) coated with polydimethylsiloxane (50/30-μM thickness, 1 cm) was used to absorb the volatile compounds. The fiber was desorbed at 250 °C for 3 min in splitless injection mode in an Agilent gas chromatograph series 7890A fitted with an Agilent HP-5MS fused silica capillary column (30 m long × 0.25 mm ID × 0.25 μm film thickness) coupled to a 5975C mass spectrometer detector (Agilent). The temperature conditions were adjusted as follows: 40 °C for 2 min, raised at 10 °C/min to 150 °C, then at 15 °C/min to 220 °C with holding for 5 min; injector temperature was 250 °C. The total run time was 23 min with helium as the carrier gas adjusted to a flow rate of 0.794 mL/min in splitless mode, with ionization energy of 70 eV. Compounds were tentatively identified using the NIST mass spectral library (Version 5) with Chemstation version E.02.00.493. MS description was based on the NIST library, Version 05 (Agilent). Volatile identification was based on the RI and mass spectra as indicated. Specific compounds were also identified by authentic standards. Relative quantification was done according to the retention time of each compound.

### 4.10. Ethylene and Respiration

Red and green mango fruit cv. Shelly were stored at 5, 8 or 12 °C. Eight red-colored and eight green-colored fruit were enclosed in glass jars (one fruit per jar). The respiration production rates of the mango fruit were measured by closing the bottles for 1 h. Samples were taken using syringes and subsequently analyzed by GC for CO_2_ (GC-2014 gas chromatograph, Shimadzu, Tokyo, Japan) and for ethylene (Varian Model 3300 GC, Agilent Technologies, Santa Clara, CA, USA). The respiration rate was measured after harvest, and after 1 week and 3 weeks in cold storage.

### 4.11. Statistical Analysis

Data were analyzed for significance of differences by Student’s t-test or ANOVA with Tukey–Kramer HSD using JMP software (SAS, Cary, NC, USA). *p* < 0.05 was considered statistically significant.

## Supplementary Materials

The following are available online at http://www.mdpi.com/1420-3049/23/7/1832/s1. Figure S1–S6 and Table S1.
