# Supplementary material for: Improved Cold Tolerance of Mango Fruit with Enhanced Anthocyanin and Flavonoid Contents
Source: Molecules. 2018 Jul 23;23(7):1832. doi: 10.3390/molecules23071832 (PMC6100212; doi:10.3390/molecules23071832)
Supplement: Supplementary file 1 [file molecules-23-01832-s001.pdf]

Improved cold tolerance of mango fruit with enhanced anthocyanin and flavonoid contents

Pradeep Kumar Sudheeran., Oleg Feygenberg, Dalia Maurer, Noam Alkan*

Department of Postharvest Science of Fresh Produce, Agricultural Research Organization, Volcani Center, P.O. Box 15159, HaMaccabim Road 68, Rishon LeZion 7505101, Israel

* Corresponding author: Noam Alkan, noamal@agri.gov.il

Supplementary Materials:

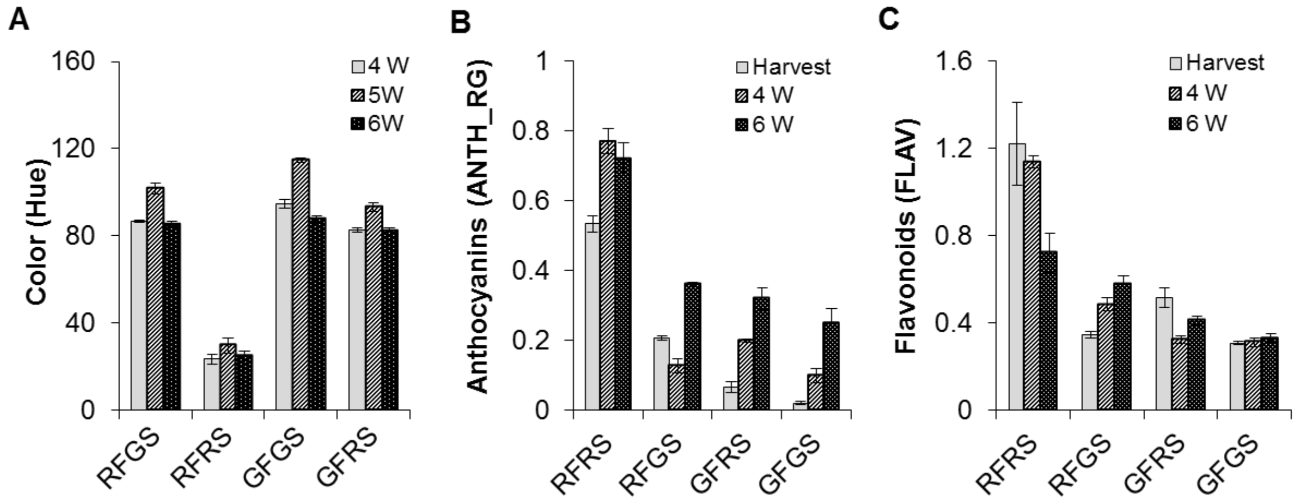

Figure S1. Color, anthocyanins and flavonoids in 'Shelly' mango fruit. Quantification of color, anthocyanins and flavonoids in the peel of red mango fruit (RF) or green fruit (GF) on their red side (RS) or green side (GS) after harvest and 4, 5 or 6 weeks (W) of cold storage at 10°C. (A) Color (hue). (B) Anthocyanin (ANTH_RG). (C) Flavonoids (FLAV).

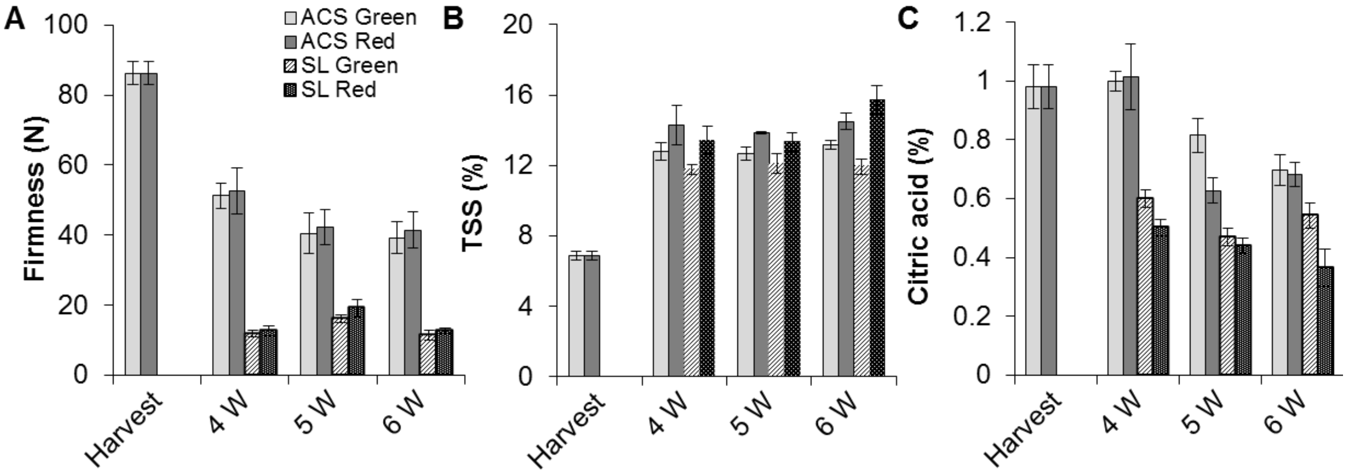

Figure S2. Evaluation of ripening parameters in green or red mango fruits stored at 10°C for 4, 5 or 6 weeks (W). (A) Fruit firmness (Newton). (B) Brix (%TSS). (C) Acid (% citric acid equivalence). ACS, after cold storage; SL, shelf life.

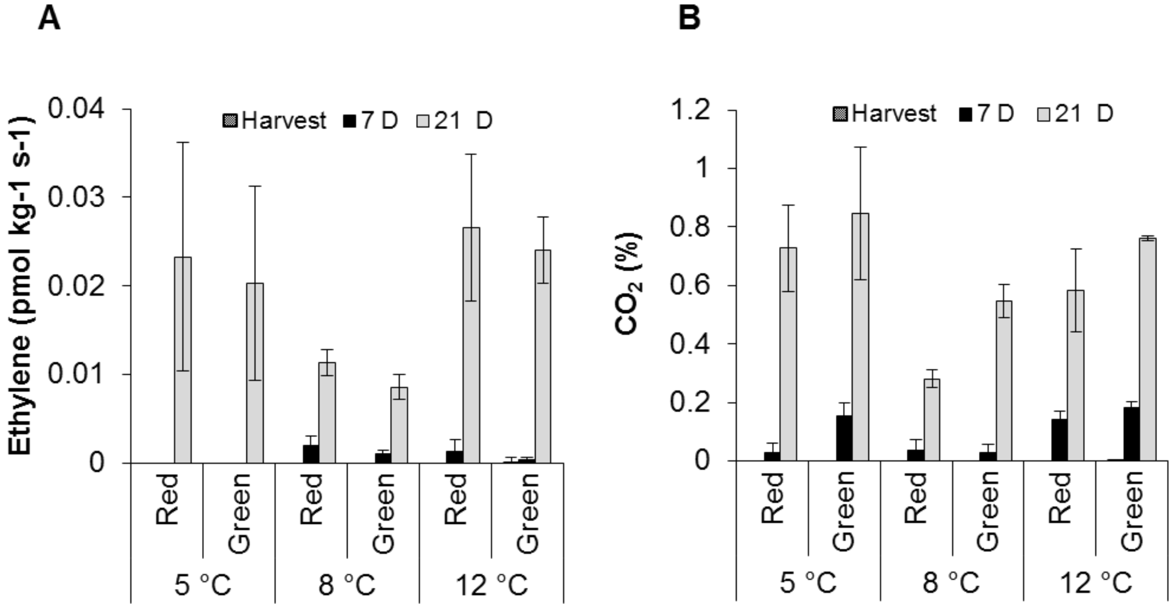

Figure S3. Ethylene and CO_2 emission from red and green mango fruit after harvest and after 7 and 21 days (D) of cold storage at 5°C, 8°C or 12°C. (A) Ethylene concentration (pmol kg^{-1} s^{-1}). (B) CO_2 concentration (%).

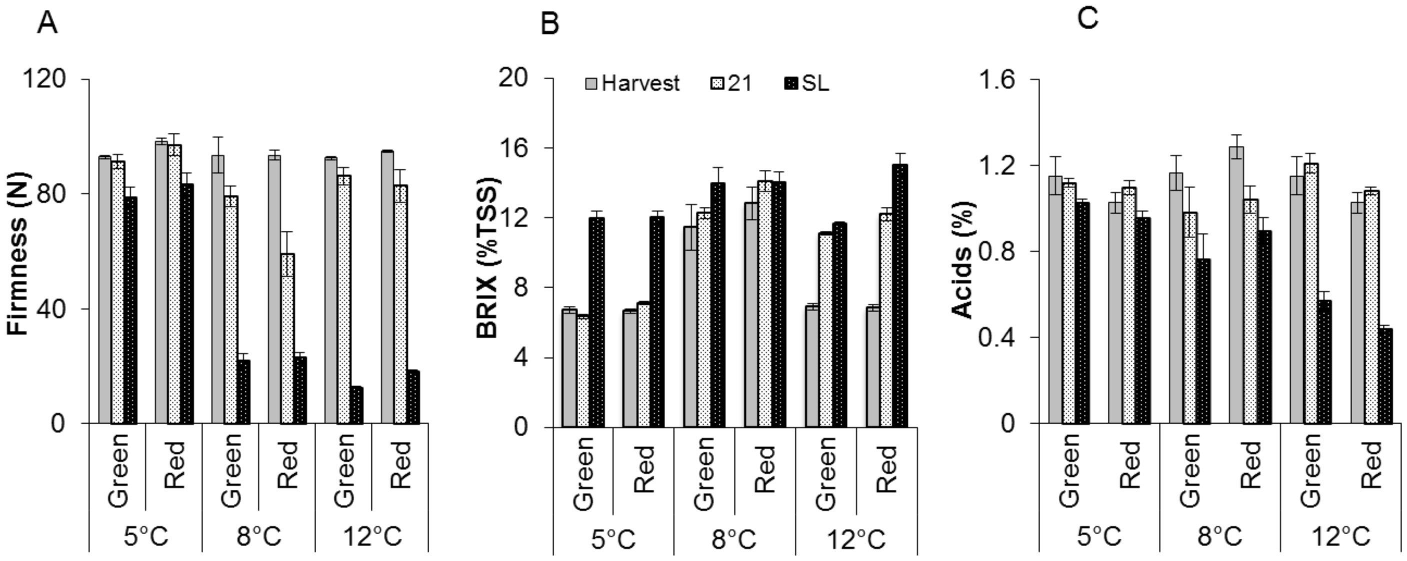

Figure S4. Ripening parameters of red and green 'Shelly' mango fruit after harvest, cold storage (5°C, 8°C or 12°C) (21) and additional shelf life (SL). (A) Fruit firmness (Newton). (B) Brix (%TSS). (C) Acid (% citric acid equivalence).

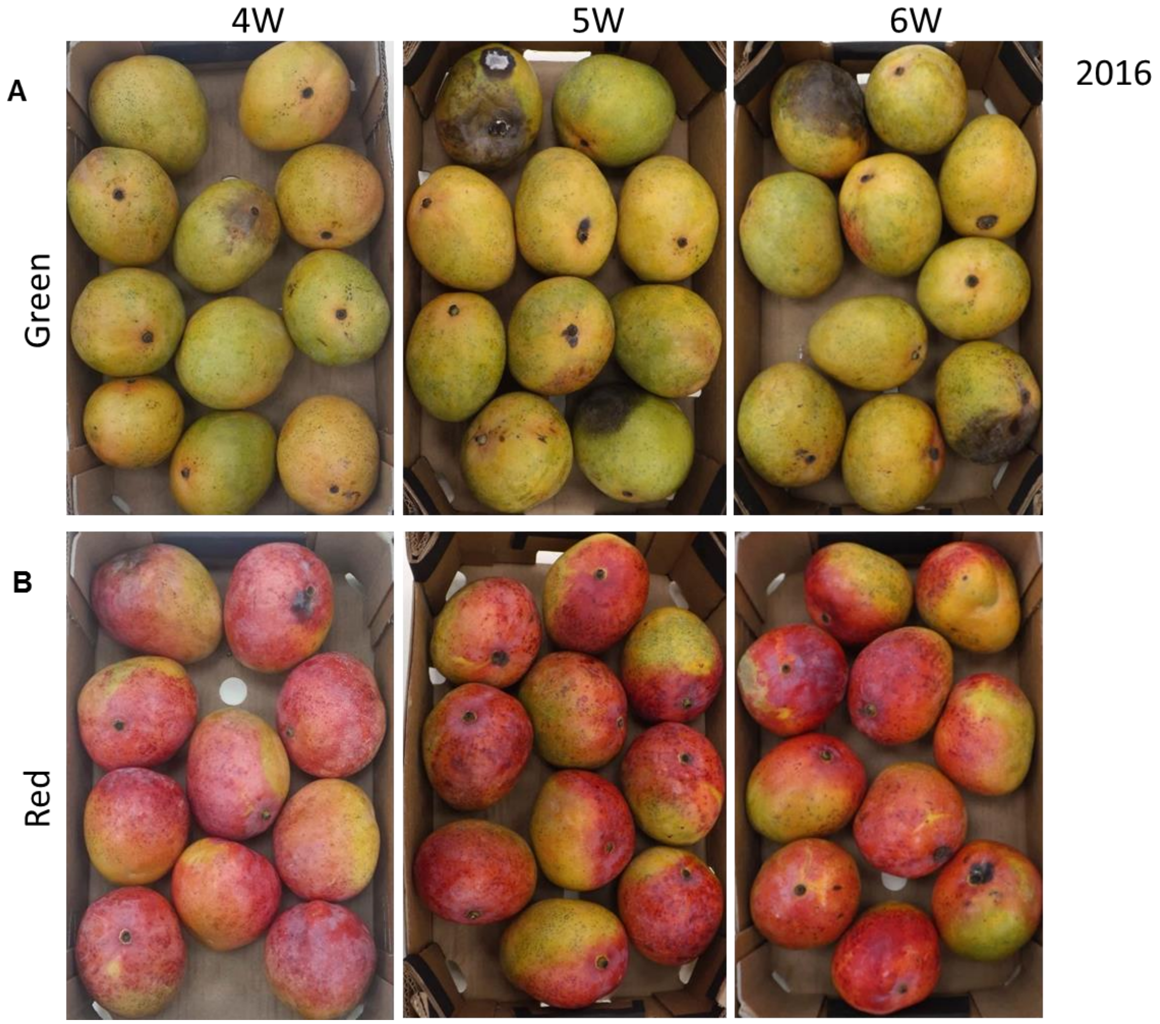

Figure S5. Representative pictures of 'Shelly' mango fruit after cold storage for 4, 5 or 6 weeks (W) and additional shelf life (2016). (A) Green fruit. (B) Red fruit.

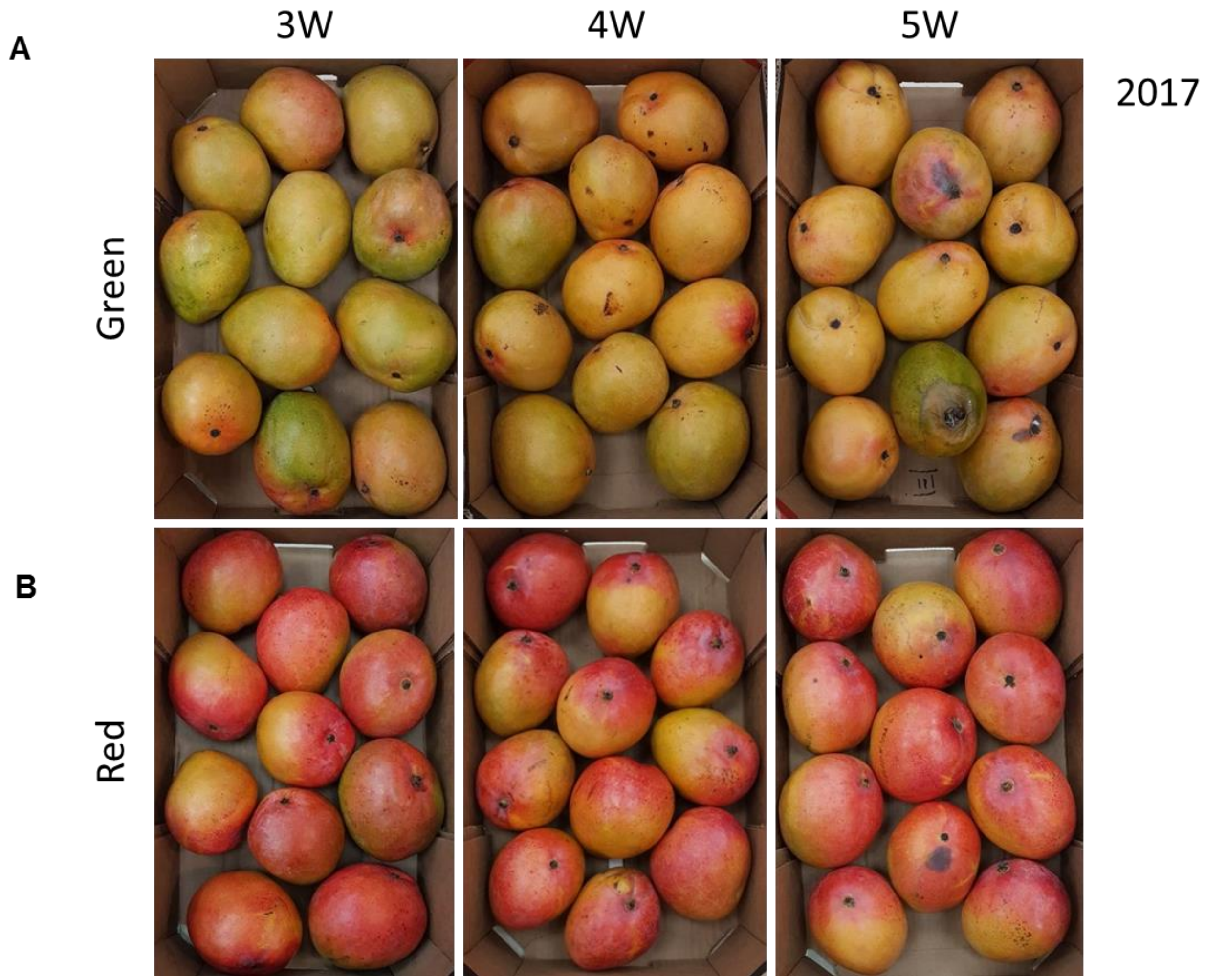

Figure S6. Representative pictures of 'Shelly' mango after cold storage for 3, 4 or 5 weeks (W) and additional shelf life (2017). (A) Green fruit. (B) Red fruit.

Table S1. Putative volatile compounds in the peel of red and green mango cv. Shelly. RT, retention time; RI, retention index.

S.NO.	List of volatile compounds	RT	RI	Red and green peels average (Conc. s^{-1})
1	(E)-2-Hexenal	6.3569	853	2.719421
2	α-Thujene	8.693	855	36.75001
3	Camphene	9.127	932	1.207285
4	6-Methyl-5-hepten-2-1	10.006	983	37.97961
5	Yomogi alcohol	10.468	997	12.50073
6	p-Cymene	11.325	1021	39.11379
7	1,8-Cineole	11.692	1025	7.004417
8	(Z)-b-Ocimene	11.7197	1032	39.84676
9	y-Terpinene	12.632	1035	130.8972
10	p-Mentha-2,4(8)-diene	13.505	1062	17.69942
11	d-Elemene	13.6	1087	9.73237
12	Eugenol	20.7708	1089	0.961743
13	β-Bourbonene	21.5051	1339	12.55311
14	β-Elemene	22.2783	1384	1.404967
15	Methyleugenol	22.5009	1402	17.93245
16	β-Caroyophyllene	23.352	1420	25.27156
17	β-Copaene	24.626	1420	29.20637
18	Drima-7,9(11)-diene	23.513	1430	2.41431
19	Germacrene D	24.6259	1469	1.846657
20	Epi-cubebol	24.9096	1480	3.085554
21	a-Muurolene	25.533	1493	6.392239
22	Cadina-1,4-diene	26.295	1499	2.001098
23	p-Methylanisole	11.2023	1021	23.38957
24	Viridiflorene	26.2143	1494	0.149823
25	Limonene	11.5695	1032	0.334137
26	(E)-β-Damascenone	22.2727	1384	1.00241
27	1,7-Di-epi-a-cedrene	22.7011	1399	0.334137
28	n-Heptanal	7.7031	899	0.200482
